# Proinflammatory Cytokine Response and Viral Replication in Mouse Bone Marrow Derived Macrophages Infected with Influenza H1N1 and H5N1 Viruses

**DOI:** 10.1371/journal.pone.0051057

**Published:** 2012-11-30

**Authors:** Renee W. Y. Chan, Connie Y. H. Leung, John M. Nicholls, J. S. Malik Peiris, Michael C. W. Chan

**Affiliations:** 1 Centre of Influenza Research and School of Public Health, LKS Faculty of Medicine, The University of Hong Kong, Pokfulam, Hong Kong SAR, China; 2 Department of Pathology, The University of Hong Kong, Queen Mary Hospital, Pokfulam, Hong Kong SAR, China; 3 HKU-Pasteur Research Centre, Hong Kong SAR, China; INSERM, France

## Abstract

The pathogenesis of human influenza H5N1 virus infection remains poorly understood and controversial. Cytokine dysregulation in human infection has been hypothesized to contribute to disease severity. We developed *in vitro* cultures of mouse bone marrow derived macrophages (BMDMΦ) from C57BL/6N mouse to compare influenza A (H5N1 and H1N1) virus replication and pro-inflammatory cytokine and chemokine responses. While both H1N1 and H5N1 viruses infected the mouse bone marrow derived macrophages, only the H1N1 virus had showed evidence of productive viral replication from the infected cells. In comparison with human seasonal influenza H1N1 (A/HK/54/98) and mouse adapted influenza H1N1 (A/WSN/33) viruses, the highly pathogenic influenza H5N1 virus (A/HK/483/97) was a more potent inducer of the chemokine, CXCL 10 (IP-10), while there was not a clear differential TNF-α protein expression pattern. Although human influenza viruses rarely cause infection in mice without prior adaption, the use of *in vitro* cell cultures of primary mouse cells is of interest, especially given the availability of gene-defective (knock-out) mice for specific genes.

## Introduction

Ferret, rather than mouse is the experimental model of choice for studying influenza viruses, as many human seasonal influenza viruses do not infect or cause disease in mice without prior adaptation. However, because of the extensive availability of immunological reagents and the fact that mice are with a range of specific gene defects (knock-out mice); they remain an important animal model for investigating influenza pathogenesis. Many highly pathogenic avian influenza (HPAI) viruses including the current influenza H5N1 viruses do replicate in mice without prior adaptation. Human H5N1 cases continue to be reported in the Asian countries including Cambodia, China, Indonesia, Thailand, and Vietnam and in Egypt [1], [2]. All of them have coincided with outbreaks of highly pathogenic H5N1 avian influenza in poultry. The overall death rate of H5N1 patient ranges from 33% in Hong Kong in 1997 up to approximately 60% in recent outbreaks [3]. Although such case fatality estimates may be skewed by case ascertainment biased to more severely ill patients, it is clear that HPAI H5N1 disease is associated with unusual virulence for humans. Despite its inability to transmit efficiently from human to human, H5N1 virus remains one with significant pandemic concerns, not only because of its inevitability to start a pandemic but also to the disease severity of such event [1]. Therefore a better understanding of its pathogenesis is of high priority.

Human influenza A viruses have been previously reported to induce keratinocyte-derived chemokine (CXCL1), interleukin 1β (IL-1β), IL-6 and RANTES (regulated on activation, normal T cell expressed and secreted) *in vivo* in the lung of Balb/c mice [13], [14]. Our previous studies on human airway epithelial cell [4], [5] and peripheral blood derived macrophages [6] have reported that H5N1 viruses are more potent in inducing the release of pro-inflammatory cytokine and chemokine, when compared to H1N1 virus. Large quantities of type I interferon (IFN), tumor necrosis factor-alpha (TNF-α), IL-1, IL-6 and mononuclear cell attracting chemokine (CCL3/MIP-1α, CCl4/MIP-1β, CCL5/RANTES, CXCL10/IP-10) were also detected after influenza A virus inoculation of human, rat and mouse macrophages cell line [15]–[18]. These studies of innate immune responses upon influenza virus infection in human were performed in different cell types and suggested that hyper-induction of cytokines plays an crucial role in the pathogenesis of human H5N1 disease. However, more studies have found that, human macrophages of different origins, resting alveolar macrophages and peripheral blood monocyte derived macrophages [7], [8], with different methods of differentiation [9] would differ in influenza virus permissiveness and host response profile. Previous *in vivo* inbred mouse studies [10]–[12] also shed some light on the H5N1 pathogenesis, however, the response and the interaction between individual cell types and H5N1 influenza virus were not yet studied. Therefore, it is important to characterise the mouse macrophages as an experimental model in terms of permissiveness and host response profile upon the infection of different influenza virus subtypes.

In this study, we evaluated the permissiveness and pro-inflammatory cytokine and chemokine responses to influenza H1N1 and H5N1 viruses in C57bl/6N mouse isolated BMDMΦ *in vitro*. It is shown that both influenza H1N1 and H5N1 viruses infected these cells, but only the H1N1 viruses had showed evidence of releasing infectious virus from infected macrophages. In comparison to human influenza H1N1 (A/HK/54/98) virus or mouse adapted influenza H1N1 virus (A/WSN/33), influenza H5N1 (A/HK/483/97) virus was a more potent inducer of the chemokine CXCL 10 (IP-10) but there was no clear pattern in regard to expression of TNF-α protein.

**Figure 1:**
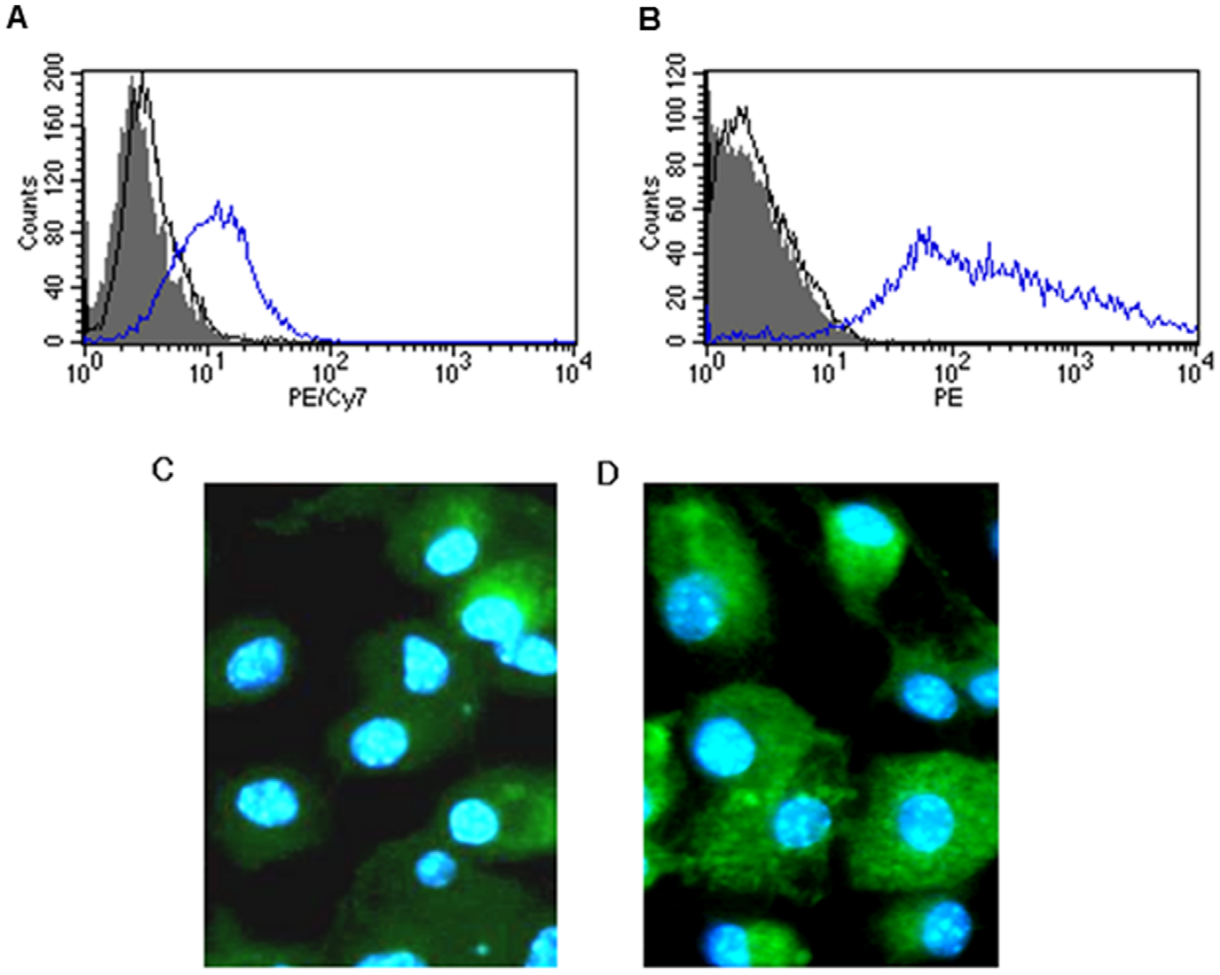
Cell characterization and lectin profile of the mouse bone marrow derived macrophages. Histogram showing the percentage of positive stained mouse bone marrow derived macrophages by flow cytometry (open peak-blue line). Isotype control (open peak-black line) and non-stained cells as negative control (shaded peak) of bone marrow derived macrophages stained with (A) CD14 and (B) F4/80. Lectin immune-staining assay to determine the sialic acid (SA) distribution on mouse bone marrow derived macrophages. (C) *Maackia amurensis* lectin (MAA) conjugated with FITC (the lectin that binds SA-α2,3Gal linked sialic acid) and (D) with *Sambucus nigra* lectin (SNA) conjugated with FITC (the lectin that binds SA-α2,6GalNAc).

## Materials and Methods

### Viruses

The viruses investigated were an influenza virus isolated from a patient with fatal influenza H5N1 disease in Hong Kong in 1997, A/Hong Kong/483/97 (483/97) (H5N1 clade 0); a virus isolated from a patient with fatal H5N1 disease in Vietnam in 2004, A/Vietnam/3046/04 (3046/04) (H5N1 clade 1), a human seasonal influenza H1N1 virus, A/Hong Kong/54/98 (54/98) and a mouse adapted influenza H1N1 virus, A/WSN/33 (WSN/33). Viruses were initially isolated and seed virus stocks were prepared in Madin-Darby canine kidney (MDCK) cells. Virus infectivity was titrated to determine tissue culture infection dose 50% (TCID_50_) in MDCK cells. The influenza H5N1 virus used in this study was handled in a Bio-safety level 3 (BSL-3) facilities in the Centre of Influenza Research, School of Public Health, The University of Hong Kong.

**Figure 2:**
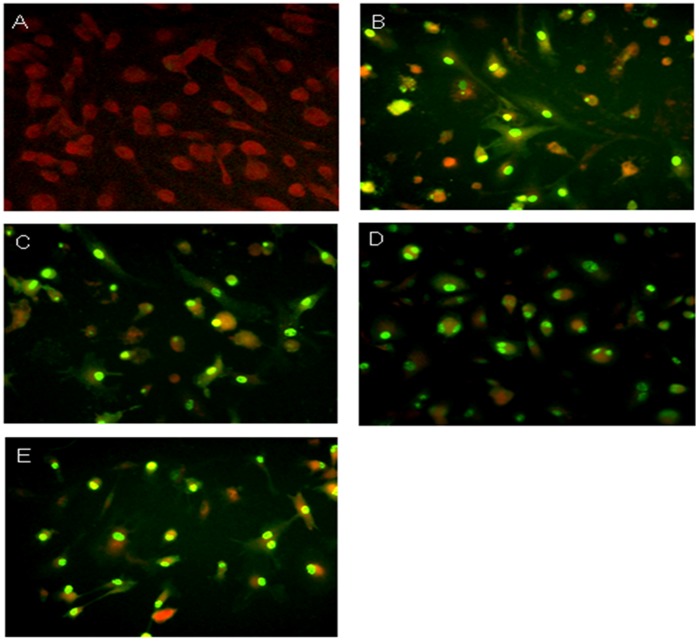
Representative immunofluorescence staining of mouse bone marrow derived macrophages after (A) Mock; (B) A/WSN/33; (C) A/HK/54/98 (H1N1); (D) A/HK/483/97 (H5N1) and (E) A/VN/3046/04 (H5N1) influenza A viruses infection. FITC-conjugated mouse antibodies (DAKO Imagen, Dako Diagnostics Ltd, Ely, UK) reacting with influenza virus matrix and nucleoprotein was used and viewed in an immunofluoresecent microscope. Mouse bone marrow derived macrophages at 20 hours post-influenza virus infection is shown.

### Mouse Bone Marrow Derived Macrophages (BMDMΦ)

Female C57bl/6N mice, 6 to 8 weeks old (Laboratory Animal Unit of The University of Hong Kong) were sacrificed using cervical dislocation before macrophage extraction under a study approved by the committee on the Use of Live Animals in Teaching and Research (CULATR) of the University of Hong Kong. Cell extraction, isolation and cultivation were performed in Bio-safety level 2 (BSL-2) cabinets to minimize possible bacterial contamination of cell cultures. Both edges of the femurs were cut and a 25-G needle was used to flush out the marrow. The marrow plug was then dispersed into single cells and was centrifuged at 400 g for 5 min. Gey’s solution in ice was used to lyse the erythrocytes in the cell suspension for 4 minutes and an equal volume of RPMI-1640 medium with 5% FCS was added prior to centrifugation. The cell pellet was then washed with warm RPMI-1640 at least twice and then resuspended in the RPMI-1640 medium supplemented with 5% FCS, 100 units/mL penicillin and 100 µg/mL streptomycin, 1.3µg/mL Amphotericin B (Cambrex Bio Science, Walkersville, Inc., Maryland, USA) and 6 ng/mL recombinant Macrophage Colony Stimulating Factor (M-CSF, R&D systems). The cells were seeded at a density of 5×10^5^ cells/ml in bacteriologic grade petri-dishes for 14 days to allow differentiation. After the trypan-blue exclusion test, viable cells were seeded into tissue culture grade 24-well plates at a density of 2×10^5^cells/ml on coverslips. Purity of MΦ was confirmed by flow cytometry (FACSSCalibur; Becton Dickinson). A 1∶50 dilution of fluorescein isothiocyanate (FITC) conjugated rat anti-mouse CD14 and F4/80 antibodies (eBioscience, San Diego, CA, USA, 24°C, 45 min) were used. The FITC-stained cells were detected by measuring green light emitted at 530 nm (FL1 channel).

**Figure 3:**
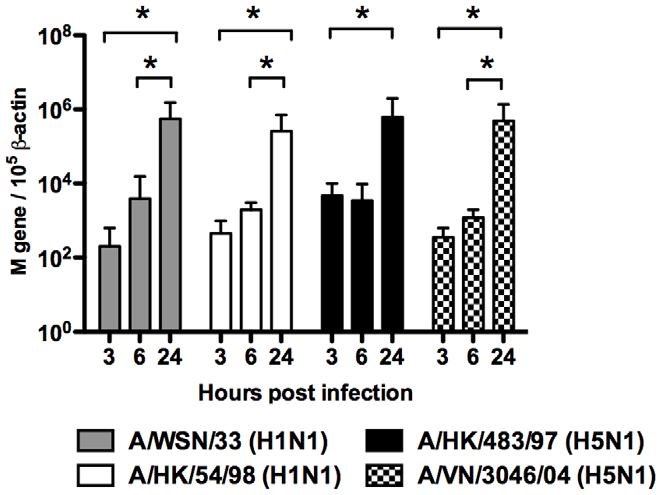
Viral matrix (M) gene expression copy number normalized to β-actin gene expression (10^5^ copies) by quantitative RT-PCR in influenza virus infected mouse bone marrow derived macrophages. Matrix gene mRNA copy number was assayed 3 h, 6 h and 24 h post-infection and normalized to those of β-actin mRNA in the corresponding sample. Means of triplicate assays are shown with standard error. Asterisk indicates statistical difference (*p*<0.05).

**Figure 4:**
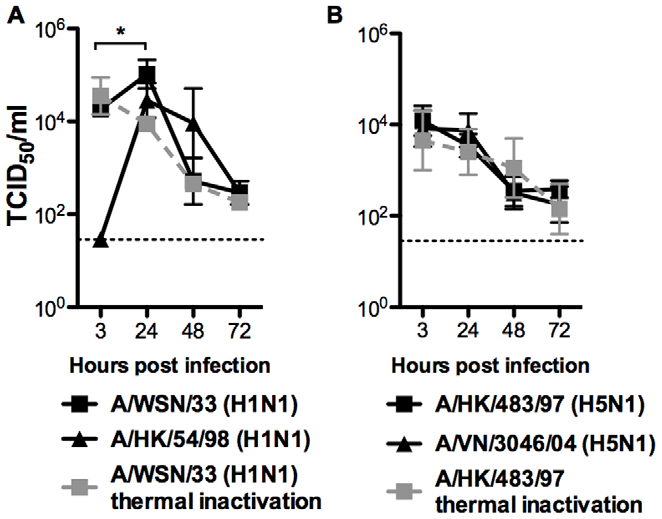
Virus titer detected in the supernatant of influenza A virus infected mouse bone marrow derived macrophages. Virus titer of various (A) influenza H1N1, and (B) H5N1 influenza viruses was determined at 3, 24, 48 and 72 h post-influenza virus infection of mouse bone marrow derived macrophages. Means and standard error of triplicate assays were shown. Dotted line represents the lowest detection limit of the TCID_50_ assay. The thermal inactivation (serial dilution of influenza virus was incubated in the cell-free culture medium alone at the corresponding time points) curves (dotted line) of influenza H1N1 and H5N1 viruses at 37°C were determined from culture wells without macrophages.

### Flow Cytometry

BMDMΦs was detached from the culture dish using cold 1×PBS with 20 mM EDTA. The detached cells were washed once with PBS and centrifuged at 400 g for 5 minutes. The cells were then incubated in PBS supplemented with 0.1 g/100 ml bovine serum albumin solution with 10% FCS for 30 minutes at room temperature and pelleted by centrifugation at 400 g for 10 minutes. The pellet was then resuspended in 100µl of PBS supplemented with 0.1 g/100 ml bovine serum albumin solution with 10% FCS. 10µl of PE-Cy7-labeled anti-mouse-CD14 antibody and PE labeled anti-mouse-F4/80 staining were used in the case of dual staining (eBioscience, San Diego, CA, USA). The respective antibody and cells were mixed and incubated for 45 minutes at room temperature. The cells were then washed once with 1×PBS and analyzed using flow cytometer. Cell suspension without the addition of antibodies and their corresponding isotype-control antibodies (PE-Cy7 conjugated IgG1 and PE conjugated IgG1 (eBioscience) were used as negative control.

**Figure 5:**
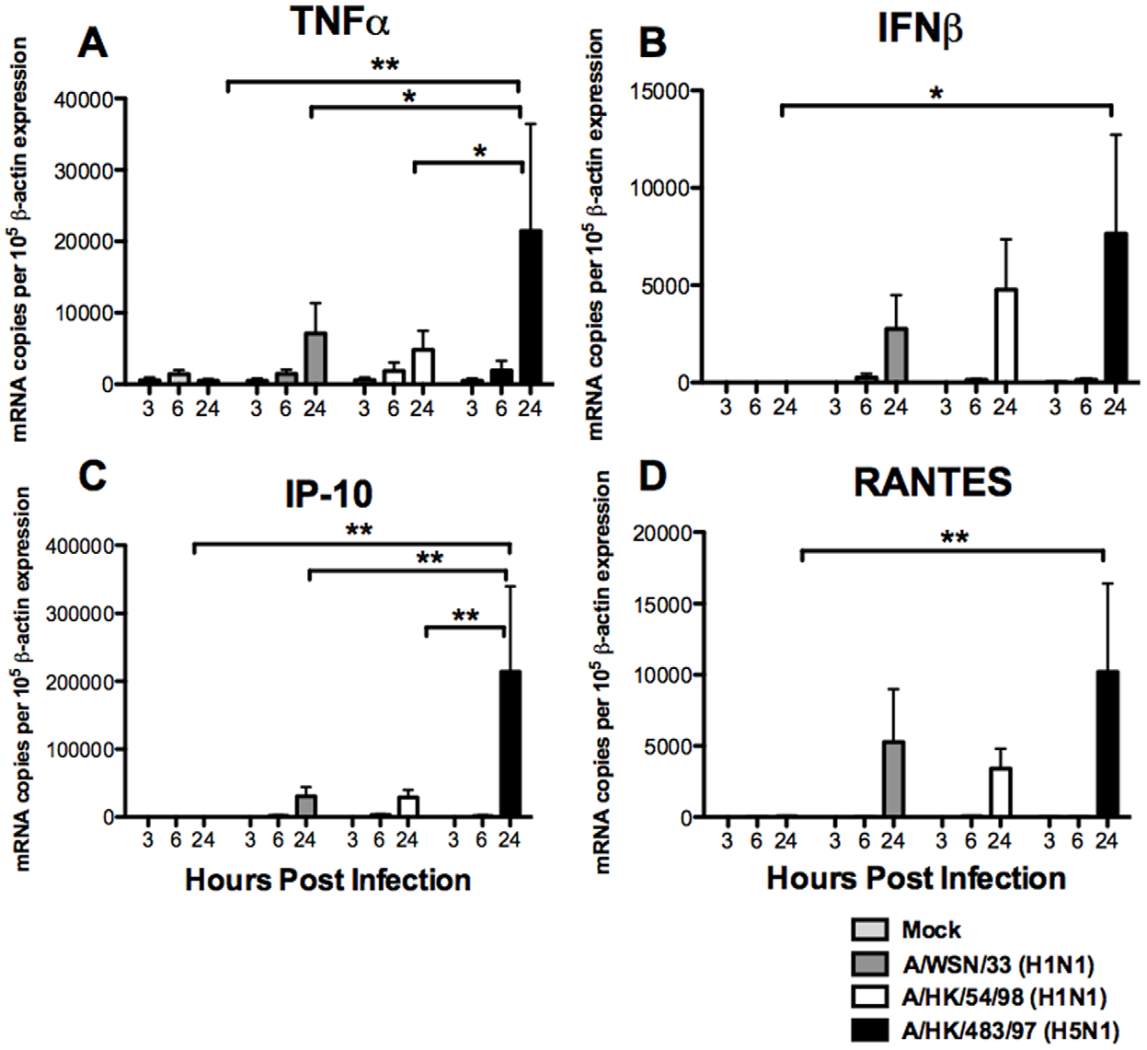
Cytokine and chemokine gene expression in mouse bone marrow derived macrophages after influenza A virus infection. The cytokines **(**A) TNF-α, (B) IFN-β, (C) IP-10 (CXCL-10) and (D) RANTES (CCL-5) mRNA gene expression profile of influenza-virus-infected mouse bone marrow derived macrophages were analyzed by quantitative RT-PCR. The graph shows the mean and the standard error from three independent experiments. Single and double asterisks indicate statistically significant difference with *p*<0.05 and *p*<0.01 respectively.

### Lectin Immunofluorescence Assay

BMDMΦs monolayer was fixed using 4% paraformaldehyde for 1 hour and washed with PBS. 0.1 M Tris buffer at pH 7.4 with 150 mM NaCl (TBS) was used to wash the cells for three times. The cells were then incubated with 1∶100 FITC conjugated *Sambucus nigra* lectin (SNA-I) (EY laboratories, Inc. R-6802-1) and 1∶100 FITC conjugated *Maackia amurensis* lectin (MAA) (EY laboratories, Inc. F-7801-2) diluted with 0.1 M TBS for 1 hour at room temperature in dark. After incubation, the cells were washed using 0.1 M TBS for three times and the nuclei of the cells were stained using 5 µg/ml DAPI for 4 minutes. The cells were washed again with 0.1 M TBS for three times and the coverslips were mounted with DAKO fluorescent mount (Dako, S3023).

**Figure 6:**
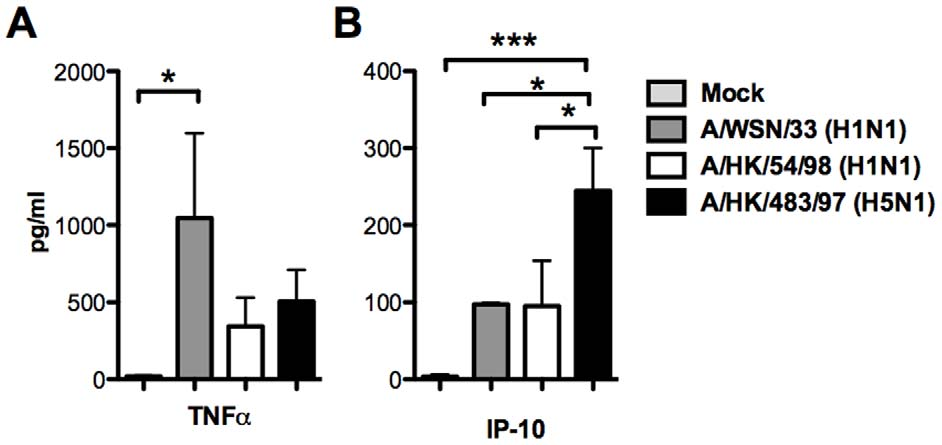
Cytokine and chemokine secretion from mouse bone marrow derived macrophages after influenza A virus infection. (A) TNF-α (B) IP-10 protein secreted by the mouse bone marrow derived macrophages after influenza A viruses infection (as denoted in legend). Mean and standard error of duplicate assays are shown. All influenza A virus infected mouse macrophages secrete significantly higher concentration of TNF-α than mock infected cells (*p*<0.05).

### Influenza Virus Infection of BMDMΦ

Mouse BMDMΦs in 24-well tissue-culture plate was infected at a multiplicity of infection (MOI) of two unless otherwise indicated. After 1 hour of virus adsorption, the virus inoculum was removed and the cells were washed with warm culture medium. After 20 hours of infection, the cell monolayer was fixed with 4% paraformaldehyde. Evidence of viral infection was established by (a) assaying viral matrix RNA after infection by quantitative RT-PCR, (b) viral antigen expression by immunofluorescence staining with mouse anti-influenza nucleoprotein and matrix antibody conjugated with FITC (DAKO Imagen, Dako Diagnostics Ltd, Ely, UK) and (c) assaying infectious virus in cell culture supernatant by TCID_50_ assay to demonstrate complete virus replication.

### Viral titration by TCID_50_ Assay

A confluent 96-well tissue culture plate of MDCK cells was prepared one day before the virus titration (TCID_50_) assay. Cells were washed once with PBS and replenished with serum-free MEM medium supplemented with 100 units/ml penicillin and 100 µg/ml streptomycin and 2 µg/ml of supernatant. Serial dilution was performed (from 0.5 log_10_ to 7 log_10_ dilution) and the virus dilutions were added onto the plates in quadruplicate. The plates were observed for cytopathic effect daily. The end-point of viral dilution leading to CPE in 50% of inoculated wells was estimated using the Karber method [19].

### Quantification of Cytokine mRNA by Real-time Quantitative RT-PCR

DNase-treated total RNA was isolated by means of RNeasy Mini kit (QIAGEN, Hilden, Germany). The cDNA was synthesized from mRNA with poly(dT) primers and Superscript III reverse transcriptase (Life Technologies, Rockville, MD, USA) and quantified by real-time PCR analysis with a LightCycler (Roche, Mannheim, Germany). The mRNA for tumor necrosis factor alpha (TNF-α), interferon beta (IFN-β), CXCL-10 (IFN-gamma-inducible protein-10, IP-10) and CCL5 (Regulated on Activation, Normal T Expressed and Secreted, RANTES) were quantified using real-time RT-PCR. The oligonucleotide primers and methods used for real-time quantification of mouse cytokines, viral matrix gene and the housekeeping gene product β-actin mRNA have been described previously by others [20]–[22] and our group [4]–[6], [23], [24].

### Quantification of Cytokine Proteins by ELISA

The concentrations of TNF-α, IP-10 and interferon-beta proteins in the mouse MΦs supernatants were measured by a specific ELISA assay (R&D Systems, Minneapolis, MN, USA). Samples of culture supernatant were irradiated with ultraviolet light (CL-100 Ultra Violet Cross linker) for 15 minutes to inactivate any infectious virus before the ELISA assays were done. Previous experiments had confirmed that the dose of ultraviolet light used did not affect cytokine concentration as measured by ELISA (data not shown).

### Statistical Analysis

Two-tailed student *t*-test was used to compare the differences among viral titers in the influenza virus infected cell supernatants between early and late time points post-infection. The quantitative cytokine and chemokine mRNA and protein expression profile of mock, influenza H1N1 and H5N1 virus infected cells were compared using one-way ANOVA, followed by *Bonferroni* multiple-comparison test. Differences were considered significant at *p*<0.05.

## Results

### Cell Characterization and Lectin Profile of BMDMΦ

The yield of the primary culture of mouse bone marrow derived macrophages were 3.5±0.9×10^6^ cells/mouse at 93±5% cell purity as demonstrated by the expression of the macrophage specific markers CD14 and F4/80 antibodies by flow cytometry (Fig. 1A and 1B).

Lectin immunohistochemistry on the primary culture of mouse bone marrow derived macrophages showed that both lectins, MAA (Fig. 1C) (which recognizes the accepted avian influenza receptor Siaα2-3Gal) and SNA (Fig. 1D) (which recognizes the human influenza receptor Siaα2-6) bound strongly to the mouse bone marrow derived macrophages.

### Influenza Virus Infection of BMDMΦ

Previous studies have demonstrated that avian influenza viruses can infect mice intra-nasally *in vivo* [11] and human peripheral blood derived macrophages [6] and human airway epithelial cells in vitro [4], [5], [23]. We first determined whether avian and human influenza viruses could infect mouse bone marrow derived macrophages *in vitro*. The cells were infected with influenza H5N1 (483/97 and 3046/04) and H1N1 (WSN/33 and 54/98) at a MOI of 2, and the proportion of cells expressing influenza A virus protein was analyzed at 20 h post-infection by immunofluorescence assay using an antibody specific for the virus nucleoprotein and matrix proteins. Similar proportions (about 95%) of BMDMΦ infected with both influenza H5N1 and H1N1 viruses had evidence of viral antigen (Fig. 2).

There was an increase in influenza matrix gene expression from 3 hours to 24 hours post-infection with all four influenza strains (Fig. 3). However, productive replication was only observed in BMDMΦ infected with influenza H1N1 (54/98 and WSN/33) subtype (Fig. 4A) but not in influenza H5N1 (483/97 and 3046/04) subtype (Fig. 4B). WSN/33 replicated effectively and yielded the highest viral load (Fig. 4A). Thermal inactivation curves of influenza viruses at 37°C were plotted to show the virus inactivation kinetics from culture wells without cells. The difference between the virus load of the infected cultures and the inactivation curve confirmed the presence of higher (and indeed increasing) virus titers from infected cell was due to productive virus replication (Fig. 4).

### Induction of Proinflammatory Cytokine and Chemokine in BMDMΦ

We investigated the cytokine and chemokine induction profile induced by influenza H1N1 and H5N1 viruses in primary cultures of mouse bone marrow derived macrophages. The mRNA expression of TNF-α, IFN-β, RANTES, IP-10 and the housekeeping gene, β-actin was quantified using quantitative RT-PCR at 3, 6 and 24 hours post-infection. The mRNA levels of TNF-α (Fig. 5A), IFN-β (Fig. 5B), IP-10 (Fig. 5C) and RANTES (Fig. 5D) after 24 hours post-infection were significantly up-regulated by influenza H5N1 virus (483/97) when compared with the mock infected cells (with *p*<0.01 with TNF-α, *p*<0.05 with IFN-β, *p*<0.001 with IP-10 and *p*<0.01 with RANTES), H1N1 viruses infected cells (with *p*<0.05 in TNF-α and *p*<0.01 with IP-10), and the mouse adapted WSN/H1N1 virus (with *p*<0.01 with TNF-α and IP-10). There was a trend suggesting that the IFN-β and RANTES gene expressions were more induced by influenza H5N1 virus (483/97) at 24 hours post-infection when compared to that in influenza H1N1 (54/98) and mouse adaptive H1N1 (WSN/33) viruses infected mouse macrophages, but statistical significance was not achieved (Figure 5B and 5D).

Inactivation of the virus by ultraviolet irradiation prior to infection of the mouse macrophages abolished cytokine induction (data not shown) suggesting that virus replication was required for cytokine induction. Furthermore, even an increase in the MOI of influenza H1N1 (54/98 and WSN/33) viruses up to 10 did not result in the cytokine and chemokine mRNA expression level to levels similar to those induced by influenza H5N1 (483/97) virus (data not shown). The observations remained valid whether the cytokine mRNA expression data were analyzed with or without normalization for β-actin mRNA concentrations (data not shown).

### Secretion of Cytokine Proteins from BMDMΦ

We further investigated the secretion of cytokine proteins from BMDMΦ infected by influenza H1N1 and H5N1 viruses. The protein concentrations of the TNF-α, IP-10 and IFN β were measured by ELISA in culture supernatants of BMDMΦ infected by the influenza A viruses. There appeared to have discordance between TNF-α mRNA expression and the TNF-α protein secretion in infected BMDMΦ (Figure 6A). The mouse adapted influenza H1N1 virus (WSN/33) which is lethal to mice *in vivo*, induced larger amount of TNF-α secretion than mock (*p = *0.05) infected BMDMΦ. WSN/33 also induced higher levels of TNF-α than induced by influenza H5N1 (483/97) virus and human influenza H1N1 (54/98) virus infected BMDMΦ although the statistical significance was not achieved (Figure 6A). On the other hand, in parallel with the gene expression profile, influenza H5N1 virus elicited more IP-10 (CXCL-10) secretion in BMDMΦ than mock (*p = *0.001), influenza H1N1 (54/98) (*p = *0.05) and WSN/33 virus infected cells (*p = *0.05) after 24 hours post-infection. IFN-β was only detected (concentration of 48 ρg/ml) in the supernatant of influenza H5N1 virus (483/97) infected BMDMΦ at 24 hours post-infection but we failed to detect any IFN-β secreted from the supernatants of BMDMΦ after other influenza viruses infection at various time post-infection. It should be noted that the limit of detection of the mouse IFN-β ELISA was high (15.6 pg/ml) and this lack of sensitivity of the assay is likely to be responsible for this lack of detection of this cytokine in H1N1 virus infected cells.

## Discussion

The mouse is not a natural host for influenza A virus and not all strains of influenza A virus can infect and replicate productively in mice *in vivo* or in mouse macrophages *in vitro*. Therefore mouse macrophages may have some limitations as an experimental model for the study of the pathogenesis of influenza virus. Nevertheless, mouse model continues to be widely used because of its convenience and more importantly, because of the wealth of immunological reagents that are available. In addition, a full-range of gene knockout mice is available and most of them are generated in C57bl/6N mouse background, including TLR-3, TLR-4, TLR-7, TLR-8 and MYD88 knockouts. The availability of TLR family knockouts are important, as TLRs function as sensors to recognize a large variety of infectious agents and elicit subsequent innate immune response to limit further invasion [25]. Thus, the findings in regard to influenza virus susceptibility, replication kinetics and host responses in C57bl/6N derived primary cell-types is of interest and can be useful to enhance our understanding of the pathogenesis of influenza.

It remains controversial on whether influenza A viruses can replicate in mouse macrophages *in vitro*. Different researchers have reported a fully productive replication [27], a low level release of infectious progeny [28], and abortive replication [29] or an interruption of viral replication at the viral protein translation stage [30]. These discrepancies may relate to the differences in viral strains, culture methods, while the differences in susceptibility of mouse macrophages to influenza virus was suggested to be determined by genetic factors [26] and other parameters.

In our study, the influenza H5N1 virus infection of mouse bone marrow derived macrophages led to the initiation of viral gene transcription and viral protein synthesis. There was no release of progeny virus and H5N1 virus infections of mouse bone barrow derived macrophages appeared to be abortive (Figure 4B). The influenza matrix gene copy number was found to increase with time from 3 hours to 24 hours post-infection. The influenza viral matrix and nucleoprotein were expressed in >90% of infected mouse macrophages with both influenza H5N1 and H1N1 viruses (Fig. 2). These findings suggested that double-stranded RNAs were generated in influenza H5N1 virus-infected mouse macrophages. Double-stranded RNA is a potent inducer of proinflammatory cytokines, for instance, TNF-α and IFN-ß, which can trigger cell signaling pathways such as those mediated through RNA-dependent protein kinases and IFN regulatory factor 3 (IRF-3) [31]. Therefore, the accumulation of the double-stranded RNA within the H5N1 infected cell would partly explained the induction of proinflammatory cytokines and chemokine, even in the absence of productive virus replication. Therefore mouse macrophages may still be a useful model for the detailed study of the mechanisms of the H5N1 associated host responses and in particular, to investigate the effect of specific gene knock-outs on cell signaling.
